# Rosmarinic Acid as a Candidate in a Phenotypic Profiling Cardio-/Cytotoxicity Cell Model Induced by Doxorubicin

**DOI:** 10.3390/molecules25040836

**Published:** 2020-02-14

**Authors:** Qiao Zhang, Jing Li, Sha Peng, Yanling Zhang, Yanjiang Qiao

**Affiliations:** 1 School of Chinese Pharmacy, Beijing University of Chinese Medicine, Beijing 100102, China; 2 Beijing Key Laboratory of Chinese Materia Medica Foundation and New Drug Research and Development, Beijing 100102, China

**Keywords:** rosmarinic acid, phenotypic profiling, morphological pattern recognition, hiPSC-CMs

## Abstract

Advances in cancer treatment have led to significant improvements in long-term survival in many types of cancer, but heart dysfunction and heart failure, associated with cancer treatment, have also increased. Anthracyclines are the main cause of this type of cardiotoxicity. In this study, we describe a combined experimental and cell morphology analysis approach for the high-throughput measurement and analysis of a cardiomyocyte cell profile, using partial least square linear discriminant analysis (PLS-LDA) as the pattern recognition algorithm. When screening a small-scale natural compound library, rosmarinic acid (RosA), as a candidate drug, showed the same cardioprotective effect as the positive control. We investigated the protective mechanism of RosA on a human cardiomyocyte cell line (AC16) and human induced pluripotent stem-cell-derived cardiomyocytes (hiPSC-CMs). We showed that RosA pretreatment suppressed doxorubicin (Dox)-induced cell apoptosis and decreased the activity of caspase-9. RosA promotes the expression of Heme oxygenase-1 (HO-1) and reduces the production of reactive oxygen species (Ros), which is induced by Dox. Meanwhile, it can also promote the expression of cardiac-development-related protein, including histone deacetylase 1 (HDAC1), GATA binding protein 4 (GATA4) and troponin I3, cardiac type (CTnI). Collectively, our data support the notion that RosA is a protective agent in hiPSC-CMs and has the potential for therapeutic use in the treatment of cancer therapy-related cardiac dysfunction and heart failure.

## 1. Introduction

Advances in cancer treatment have led to a significant increase in the long-term survival rate of many types of cancer but have also significantly increased the adverse side effects associated with treatment [1]. In the long term, the risk of death from cardiovascular problems causes exceeds the risk of tumor recurrence [2]. Anthracyclines are the best example of this type of cardiotoxicity [3], such as aclacinomycin, daunorubicin, Dox, etc. Cardiotoxicity is characterized by irreversible myocardial damage due to the cumulative administered dose [4]. To account for their dose-dependent cardiac dysfunction, many mechanisms have been proposed, such as the production of reactive oxygen species [5,6], the accumulation of anthracycline metabolites that disrupt sarcomere structure and function [7], and mitochondrial biogenesis [8]. Although the American Heart Association (AHA) has provided proposals for preventive strategies, which include primordial prevention (initiation of cardioprotective medications after the diagnosis of cancer) and primary prevention (initiation of medical therapy with cancer treatment in patients with cardiovascular risk factors) [9], medical professionals lack novel and effective drugs to improve chemotherapy-related cardiomyopathy.

Many types of natural substances have been investigated for their cardioprotective effects [10,11]. Typically, plant phenolics have been found to mitigate anthracycline-induced toxicity in rat cardiomyocytes [12,13,14]. Caffeic acid, chlorogenic acid, and RosA have been found to have antioxidant effects against doxorubicin-induced toxicity in neonatal rat cardiomyocyte cells, and RosA was found to be the most effective cytoprotective agent [15]. RosA inhibited ADR-induced apoptosis by inhibiting ROS generation and JNK and ERK activation [16]. Interestingly, the relative protectivity of those three compounds against the lipid peroxidation of heart membranes, mitochondria, and microsomes (chlorogenic acids > RosA > caffeic acids) did not correlate with the effectiveness of these compounds in terms of cardiomyocyte protectivity (RosA > chlorogenic acids) [15]. Remarkably, these small molecules generally target multiple effectors, thereby exerting subtle pleiotropic effects (both on- and off-target) at the cellular level [17,18]. This means that simply studying the in vitro potency and/or performing single-parameter high-throughput image-based cell screening is of limited use to identify potential drug-like molecules or understand their mode of action [19]. Therefore, the rate of success during lead selection and optimization in drug discovery benefits from multiparameter phenotypic profiling [20]. Here, we describe a combined experimental and cell morphology analysis approach for the high-throughput measurement and analysis of morphological of cardiomyocyte cell profile. We applied this pattern recognition methodology to assess the cardiomyocyte phenotype changes induced by Dox, transforming 302 morphological parameters into a single score to indicate the cytoprotective ability. RosA showed the greatest cardioprotective effect among a small-scale natural compound library. The cardioprotective effectiveness of RosA has been well-recognized in rat cardiac muscle cells. However, the protective ability of RosA on human-sourced cardiomyocytes is unknown. Therefore, we investigated the effects of RosA on AC16 cells and hiPSC-CMs damaged by doxorubicin in the hope of elucidating its mode of action.

## 2. Results

### 2.1. Using a High-Content Assay to Establish a Cardiotoxicity H9C2 Cell Model

To develop the cell phenotypic analysis for the Dox-induced cardiotoxicity cell model, H9C2 cells were treated with a series of concentrations of Dox. After 24 h, the cells were stained with a mixed dye, which included tetramethyl rhodamine methyl ester (TMRM), calcein tetra acetoxymethyl ester (Calcein AM), and Hoechst 33342. Various quantitative parameters were extracted from the microscopy image by a high-content analysis instrument, including Cell Number (DAPI channel), Nuclear Area (DAPI channel), Nuclear Intensity (DAPI channel), Cytoplasm Area (FITC channel), Calcium Intensity (FITC channel) and Mitochondrial Intensity (Texas red channel; Figure S1). The results suggested that 1 μM Dox induces significant changes in multiple phenotypes. Relative to naïve group, quantitative parameters in model cells (1 μM Dox) were significantly increased (Calcium Intensity and Mitochondrial Intensity) or decreased (Cell Number, Nuclear Area, Nuclear Intensity and Cytoplasm Area; Figure S1).

### 2.2. Using Cell Morphological Parameters for Pattern Recognition

**Figure 1:**
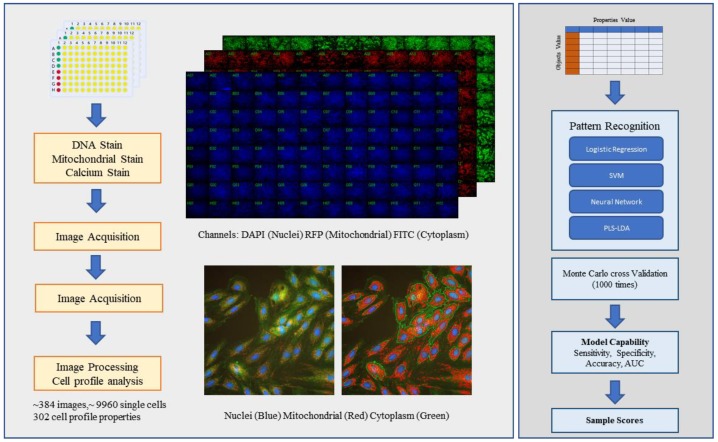
High-throughput imaging methodology. (**A**) Schematic representation of high-throughput imaging methodology. Cells are grown in flat-bottom plates and a compound is added to the desired wells. After incubation, cells are stained with the dye mix (Hoechst 33342, tetramethyl rhodamine methyl ester (TMRM), and Calcein-AM). Image acquisition is by a commercial instrument and four images were collected per channel per well. The image dataset is then subjected to image and data processing by CellProfiler. (**B**) Schematic representation of the image data analysis pipeline. Single-cell images were created for 302 cell properties. The cells treated with Dox were labeled as “positive” and the non-treatment cells were labeled as “negative.” A pattern recognition model was built by four algorithms and validated by Monte Carlo cross-validation. The sample scores were calculated by the classification model to represent the cell morphologies.

In order to characterize and analyze the cellular morphological response to Dox-induced cytotoxicity, we developed high-throughput image analysis and morphological parameter pattern recognition workflow using CellProfile [21] and MATLAB (Figure 1). In total, 302 morphological parameters were extracted from 204 objects (101 objects from naïve group and 96 objects from model group) by CellProfile^®^ for training a pattern recognition algorithm to discriminate between the naïve group and the model group (1 μM Dox).

**Figure 2:**
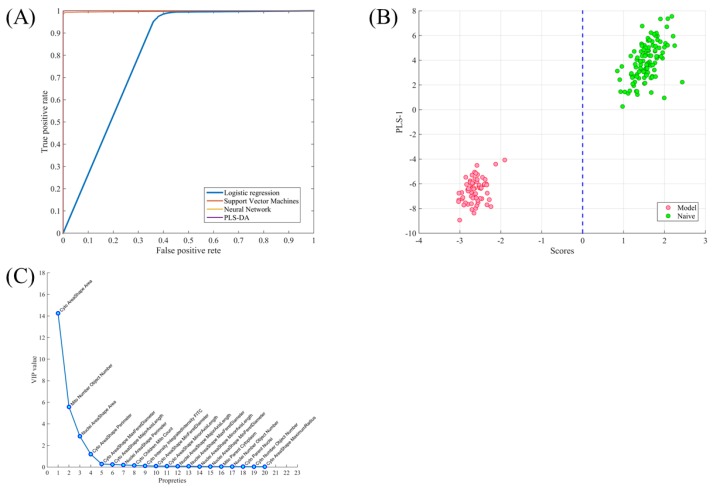
Pattern recognition of model and naïve cells. (**A**) Receiver operator characteristic (ROC) of four models. The area under the curve (AUC) of Partial Least Squares Linear Discriminant Analysis (PLS-LDA) equaled 0.999, which is the highest of all, and the AUC of LogReg equaled 0.719. (**B**) Visualization of the test samples according to the PLS-LDA scores vs. the first latent variable spaces. Data from individual images of model cells (models, red dots, *n* = 65) and naïve cells (naïve, green dots, *n* = 109) are separated along the horizontal axis. A score below 0 is classified as a model cell. (**C**) Variable importance in projection (VIP) values with cell properties. This represents the importance of each property in the trained PLS-LDA model.

To validate the classification performance of the trained pattern recognition model, Monte Carlo cross-validation (80% in training set and 20% in the testing set, 1000 times run) was used. The performance of four pattern recognition algorithms was compared (Table S1): Logistic Regression (LogReg), Partial Least Squares Linear Discriminant Analysis (PLS-LDA) [22], Least Squares Support Vector Machines (LS-SVMs) [23] and Neural Networks. The best results were obtained with the PLS-LDA model, with an area under the curve (AUC) of the receiver operator characteristic (ROC) curve of 0.999 and an accuracy of 0.9958 (Figure 2A). In the PLS-LDA model, the scores were used to represent objects in the naïve and model groups, which were then projected into model space as shown in Figure 2B. In the PLS-LDA model, the value of variable importance in projection (VIP) estimated the importance of each variable in the projection [24]. The VIP scores for cytoplasm area, mitochondrial number, nuclear area, and cytoplasm perimeter were more than 1, which indicates that the four parameters are the most obvious morphological response to Dox-induced cytotoxicity (Figure 2c).

### 2.3. Using Morphology Pattern Recognition to Assess the Cardioprotection of Natural Compounds

**Figure 3:**
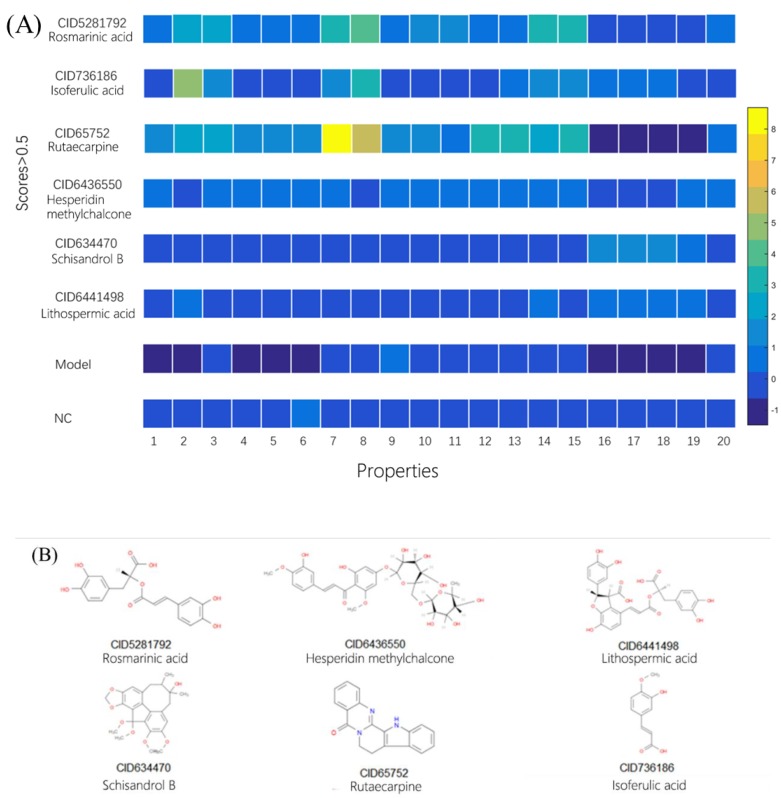
Classification of the effect of natural compounds in model cells. (**A**) Heat map representing the morphologies of model cells, naïve cells, and model cells treated with the various natural compounds (10 μM, 24 h). (**B**) Chemical structures of the natural compounds whose scores were more than 0.5.

**Figure 4:**
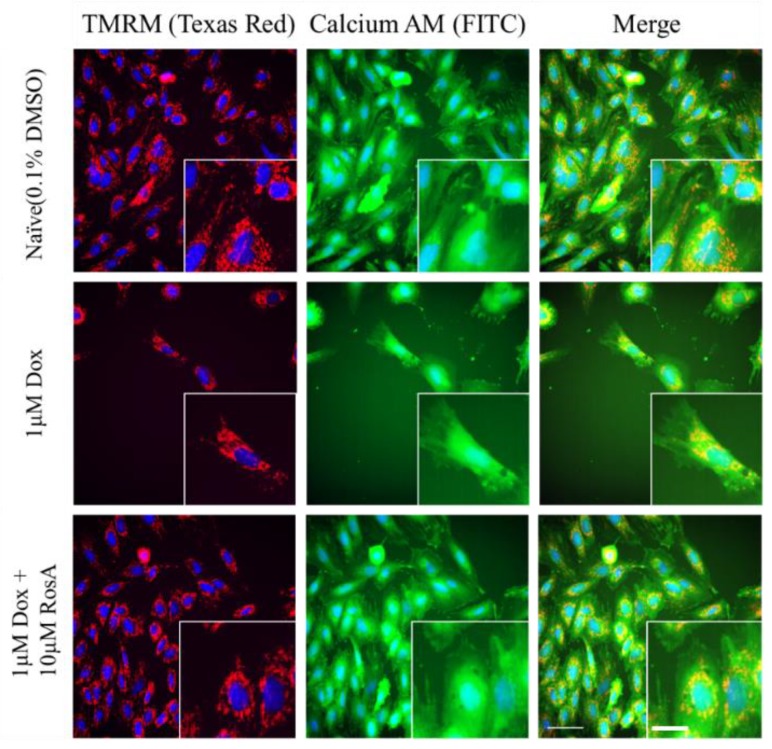
Phenotypes associated with model cells pretreated with RosA. The green fluorescence in the FITC channel is Calcein-AM, the red fluorescence in the Texas red channel is TMRM, and the blue fluorescence in the DAPI channel is Hoechst 33342.

Using the previous workflow, the Dox-induced cardiotoxicity cell model was used to evaluate the protective capacity of 88 natural compounds. The natural compounds at 10 μM were incubated with H9C2 cells for 24 h, then Dox was added to the final concentration of 1 μM and incubated for 24 h. We applied the above strategy to quantify the effects of all the natural compounds. The PLS-LDA model scores of compounds are shown in Table S2, and compounds with scores of more than 0.5 are shown in Figure 3A. VIP scores in the top 20 were used to present the protective capacity of candidates (Figure 3B). In descending order of PLS-LDA model scores, the six best candidates were CID5281792 (Rosmarinic acid), CID736186 (Isoferulic acid), CID65752 (Rutaecarpine), CID6436550 (Hesperidin methylchalcone), CID634470 (Schisandrol B) and CID6441498 (Lithospermic acid). Compared with the model group, rosmarinic acid (RosA, CID5281792) showed the best protection in the Dox-induced cardiotoxicity cell model (Figure 4).

### 2.4. RosA Protects AC16 Cells Against Dox-Induced Cell Apoptosis

**Figure 5:**
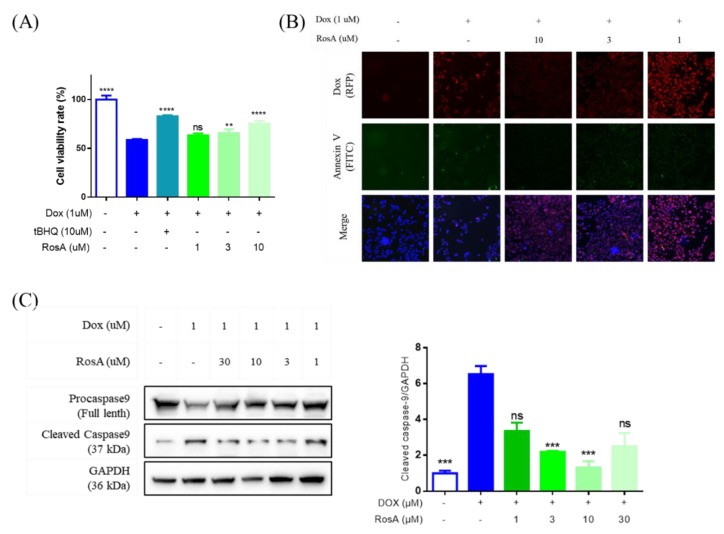
Effect of RosA on Dox-induced AC16 cell apoptosis. (**A**) The cell viability of RosA-pretreated cells after Dox treatment for 24 h. (**B**) The green fluorescence is Annexin V-FITC staining positive cells, and the red fluorescence is Dox staining positive cells. The blue fluorescence is Hochest 33342 staining positive cells. Apoptotic cells were stained by green and blue fluorescence, while normal cells were stained only by blue fluorescence. (**C**) Cleaved Caspase-9 was measured by Western blot analysis in AC16 cells after treatment. GAPDH was used as an internal control. ** *p* < 0.01, *** *p* < 0.001, **** *p* < 0.0001, ns: no significant difference. 1 μM Dox-treated was the model group; 0.25% DMSO-treated was the negative control group, compared with the model group. Results are expressed as mean ± SD (*n* = 3).

To evaluate whether RosA protects cardiomyocytes from Dox-induced cell injury, a cell viability assay was performed on AC16 cells treated with Dox at a concentration of 1 μM. Tert-butylhydroquinone (tBHQ) was assessed as an inhibitor in a Dox-induced cell injury model. We used tBHQ at 10 μM and a series of concentrations of RosA to pretreat AC16 cells for 24 h. Then, the cell viability was determined after 1 μM Dox treatment for 24 h. Compared with the Dox injury group, the group pretreated with RosA (3 μM, 10 μM) had significantly increased cell viability (Figure 5A). FITC-conjugated Annexin-V was used to assess apoptosis in cells treated with compounds. Meanwhile, the fluorescence properties of Dox made it convenient to monitor the in-cell concentration of Dox. The fluorescence intensity of Annexin V-FITC and Dox was significantly increased after stimulation with 1 μM Dox for 6 h compared with unstimulated cells. The cells pretreated with RosA (3 μM, 10 μM) were shown to prevent cell apoptosis via inhibiting the accumulation of Dox (Figure 5B). The activity of caspase-9 was measured using Western blot. Following incubation with Dox, cleaved caspase-9 was increased relative to full caspase-9, and pretreatment with RosA significantly decreased the ratio of cleaved caspase-9 induced by Dox treatment, suggesting that the protective effect of RosA in Dox-associated cardiomyocyte injury relies on caspase pathways (Figure 5C).

### 2.5. Pretreatment with RosA Results in Suppressed ROS and the Generation of HO-1

**Figure 6:**
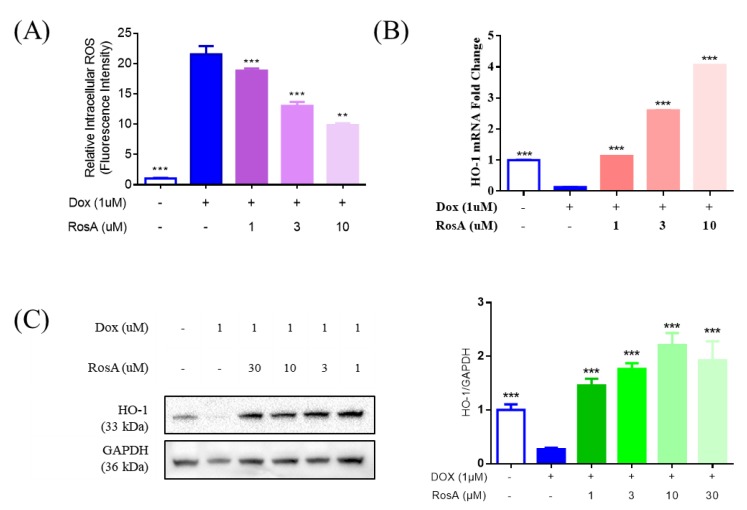
The antioxidant effect of RosA in Dox-induced AC16 injury model. (**A**) The levels of intracellular ROS were measured by DCFH-DA fluorescence. (**B**) The mRNA levels of HO-1 were evaluated using real-time polymerase chain reaction (RT-PCR), with GAPDH as the internal control. (**C**) Levels of HO-1 expression with various concentrations of RosA for 24 h as measured by Western blot, with GAPDH as an internal control. ** *p* < 0.01, *** *p* < 0.001, 1 μM Dox-treated was the model group; 0.25% DMSO-treated was the negative control group, compared with the model group. Results are expressed as mean ± SD (*n* = 3).

To further assess the cardiomyocyte protective effects associated with RosA treatment, the ROS generation was measured using DCFH-DA fluorescence probes. The treatment of 1 μM Dox in AC16 cells led to a significant increase in ROS, whereas pretreatment with RosA (at 1‒10 μM) led to a decrease compared with the Dox-injury group (Figure 6A). HMOX1 transcripts were increased after stimulation with RosA (at 1‒10 μM) compared with the controls. Furthermore, HO-1 was significantly more abundant after 1‒30 μM RosA treatment. The results also showed that treatment with Dox decreased the level of HMOX1/HO-1, which is an important antioxidative stress protein. The results suggested that RosA protects against the cardiomyocyte injury induced by Dox via antioxidative stress.

### 2.6. Pretreatment with RosA Impairs hiPS-CMs Muscle Function

**Figure 7:**
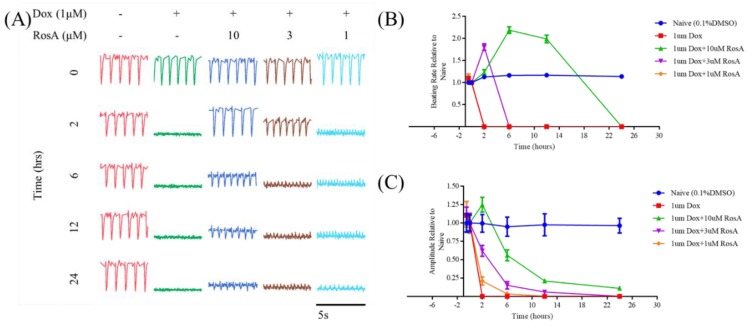
The myocardial protective effect of RosA in Dox-induced dysfunctional cardiomyocytes. (**A**) Beating patterns, (**B**) beating rates, and (**C**) amplitude of dysfunctional cardiomyocytes treated by various concentrations of RosA at selected time points (0, 2, 6, 12, and 24 h). All data were normalized by 0 h. 1 μM Dox-treated was the model group; 0.25% DMSO-treated was the negative control group, compared with the model group. Results are expressed as mean ± SD (*n* = 3).

**Figure 8:**
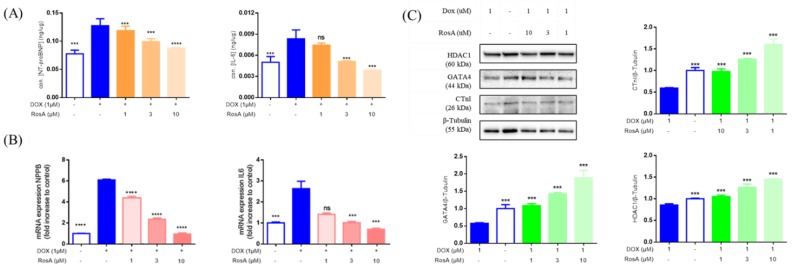
The myocardial protection of RosA in a Dox-induced hiPS-CMs injury model. (**A**) The levels of NT-proBNP and IL-6 were measured by ELISA. (**B**) The mRNA levels of NPPB and IL6 were evaluated using real-time PCR, with GAPDH as an internal control. (**C**) The levels of myocardial protection proteins, such as HDAC1, GATA4, and CTnI, were measured by Western blot, with β-tubulin as an internal control. *** *p* < 0.001, **** *p* < 0.0001, ns: no significant difference. 1 μM Dox-treated was the model group; 0.25% DMSO-treated was the negative control group, compared with the model group. Results are expressed as mean ± SD (*n* = 3).

To verify the effect of RosA protection on Dox-treated hiPS-CMs, cardiomyocyte contraction assays were performed by an RTCA cardio system. The results demonstrated that treatment with 1 μM Dox resulted in decreased rates of beating and amplitude in hiPS-CMs, as compared to those at the 0 μM Dox baseline. Pretreatment with concentrations of RosA (at 1‒10 μM) led to the maintenance of decreased rates of beating and amplitude (Figure 7). Pro-brain natriuretic peptide (NPPB), Interleukin-6 (IL6), and their mRNA were the major biomarkers of heart failure [25]. Enzyme linked immunosorbent assay (ELISA) and a RT-PCR analysis demonstrated a concentration-dependent decrease of pro-BNP and IL6, which were significantly upregulated by 1 μM Dox (Figure 8). To further study the mechanism of protection of RosA, we performed a Western blot analysis on the expression of encoding cardiac-development-related proteins [26,27,28], including HDAC1, GATA4, and CTnI. After Dox treatment, the expression of HDAC1, GATA4, and CTnI was significantly downregulated. Then, pretreatment with concentrations of RosA (at 1‒10 μM) increased the expression of cardiac-development-related proteins, including HDAC1, GATA4, and CTnI (Figure 8C).

## 3. Discussion

The case study presented here illustrates the potential of phenotypic screening assisted by objective multivariable statistical methods. Pattern recognition techniques are particularly well suited to discriminate between phenotypes that are not easily described by a few parameters and were successfully applied previously for phenotypic profiling of drug effects [29]. The latter study applied a blind approach combining 11 probes to maximally cover the cellular biology and simultaneously test the complete dose-response [17]. For the analysis of mitochondrial morphology, unsupervised learning was used to define six morphological phenotypes in CHO cells and the effects of caspase inhibition [30]. Supervised learning was used to analyze mitochondrial morphology in BEAS-2B cells treated with CI and CII inhibitors [31]. Our study differs from the above ones since it extracts all morphology parameters to express the morphology change of cardio-/cytotoxicity induced by Dox as much as possible.

To establish a representative cell model of myocardial toxicity, we determined the Dox concentration-response curve in H9C2 cells. Cell image acquisition and cell parameter calculations were implemented using high-content instruments. It was observed that treatment with 1 μM Dox-induced significant cytotoxicity, which was reflected in multiple cell parameters, including cell number, cell area, mitochondrial membrane potential, calcium ion intensity, etc. Compared with the previous study, the Dox dosage was in a reasonable range [32,33,34]. We evaluated various pattern recognition models for their ability to correctly classify model cells and naïve cells. The pattern recognition models provided excellent classifying capability; however, for a certain size of the sample, the classification capability of the model was affected by the number of samples [35]. Thus, Monte Carlo cross-validation was used to evaluate the model’s performance. When selecting AUC as the model performance measure, PLS-LDA was the best algorithm for modeling. In the PLS-LDA model, the value of VIP reflects the importance of variables, so it could be used for variable screening. Within the model, four morphology parameters were of the highest relative importance: “Cyto AreaShape Area,” “Mito Number Object Number,” “Nuclei AreaShape Area,” and “Cyto AreaShape Perimeter.” Those four morphological parameters were similar to the cell parameters calculated by the high-content instrument. Biologically, a decrease in mitochondrial object numbers indicates that the mitochondrial objects experienced damage induced by Dox.

In this study, the morphology parameters and scores of the natural product were obtained from the median of each morphology parameter and scores of the cell population which treated by the natural product. RosA has been determined to be the highest score, which indicated that it might protect myocardial cells to defense the cytotoxicity induced by Dox. The myocardial cells protect capability of RosA has been mentioned in the previous study. However, the results of the previous study were observed by rat myocardial cells [36,37]. To further verify the myocardial protective effect of RosA, AC16 cells, a human cardiomyocyte cell line [38], were used to evaluate the inhibitory effect of RosA on Dox-induced apoptosis and oxidative stress. Dox could obviously induce cell death, which causes the release of ATP. The results of a CellTiter-Glo^®^ luminescent cell viability assay indicated that RosA inhibited the process of cell death induced by Dox, for which the cell viability rate after treatment with Dox at 1 μM was similar to the cell numbers in the H9C2 cell model. Dox-induced cardiomyocyte cell apoptosis through activated caspase 9, and cell apoptosis could cause the translocation of phosphatidylserine, which could be observed by fluorescent annexin V conjugate staining. RosA at 10 μM could inhibit the caspase 9 activation and defend against the apoptotic cell formation induced by Dox at 1 μM. From the results of cell fluorescence imagery, it was found that RosA could inhibit the cumulative effect of Dox. This might be due to RosA inhibiting the increase in cell membrane permeability that is induced by Dox treatment. However, further study is needed to confirm that RosA interacts with cellular membrane structure proteins. Dox could promote the generation of Ros and inhibit the formation of HO-1. As the results suggest, RosA decreases the accumulation of Ros and increases the expression level of HOMX-1. hiPS-CMs can reflect the physiological characteristics of myocardial tissue at the cellular level and have been used in comprehensive in vitro pro-arrhythmia assay (CiPA) projects for drug toxicity evaluation [39]. Dox has a very significant influence on the physiological function of myocardial muscle, as it can inhibit the amplitude of myocardial cell pulsation and accelerate the myocardial rhythm in a short time. This phenomenon might be related to the imbalance of calcium ions in myocardial cells, which leads to calcium overload. RosA could delay the abnormal status induced by Dox. Pro-BNP and IL-6 are usually used to index heart failure in clinical studies [25,40]. In our study, RosA inhibited the increase of pro-BNP and IL-6 induced by Dox, which suggests that RosA has protective ability against the heart failure caused by Dox. The myocardial physiology was based on structural proteins and transdifferentiation proteins, such as GATA4, CTnI, and HDAC1 [26,27,28]. Our study shows that RosA increased those functional proteins’ expression, which suggests that it could protect myocardial function in the long term. In general, RosA does not completely inhibit the myocardial arrhythmia induced by Dox; however, it defended against oxidative stress and apoptosis and induced CTnI expression. This suggests that RosA could be used as a drug to treat chronic myocardial disease.

## 4. Materials and Methods

### 4.1. Chemicals and Reagents

Rosmarinic acid, which stock in 30 mM by DMSO, were purchased from Solarbio (Beijing, China). Dulbecco’s modified eagle medium (DMEM) and fetal bovine serum (FBS) were purchased from Gibco (Grand Island, NY, USA). Doxorubicin (Cat. #HY-15142A) and DCFH-DA (Cat. #HY-D0940) were purchased from MedChemExpress (Monmouth Junction, NJ, USA). PBS (PH 7.2o–7.4) was obtained from Solarbio (Beijing, China). TMRM and Calcein-AM were purchased from Molecular Probes (Eugene, OR, USA). Hoechst 33342 was purchased from Cell Signaling (Danvers, MA, USA). The natural compounds screening library, which stock in 10 mM by DMSO was supported by Yanjing Qiao laboratory.

### 4.2. Cell Cultures

H9C2 and AC16 cells (Millipore, Temecula, CA, USA) were maintained in Dulbecco’s modified Eagle’s medium (DMEM) supplemented with 10% fetal bovine serum, 100 units/mL penicillin, and 100 μg/mL streptomycin at 37 °C, 5% CO_2_. Human-induced pluripotent stem cell-derived cardiomyocytes (hiPS-CMs) were obtained from Cellapybio Co. (Cat. #2201106, Beijing, China), which were well-validated cell lines. Briefly, each well of the plates was precoated with a 1:100 diluted Matrigel solution (BD, Franklin Lakes, NJ, USA) and maintained at 4 °C overnight. The cells were incubated in a maintaining medium at 37 °C, 5% CO_2_. The cell culture medium was refreshed every two days.

### 4.3. Live-Cell Image Acquisition

H9C2 cells at a density of 10,000 per well were seeded in 96-well plates. The cells were stained by 10 nM TMRM, 50 nM Calcein-AM, and 2 μg/mL Hoechst 33342, then incubated at 37 °C for 30 min. Cells were visualized using ImageXpress Micro instruments (Molecular Devices, San Jose, CA, USA) with a 40X objective. Cell parameters were calculated by MetaMorph software (Molecular Devices, San Jose, CA, USA).

### 4.4. Cell Viability Assay

AC16 cells at a density of 10,000 per well were seeded in 96-well plates, incubated at 37 °C for 24 h, and treated with 10 μM tBHQ as a positive control and various concentrations of RosA for 24 h. Then the cells were treated with 1 μM Dox for 24 h. At the end of the experiment, cell viability was measured using a CellTiter-Glo Luminescent Cell Viability Assay (Promega, Madison, WI, USA).

### 4.5. Annexin V-FITC Staining Assay

AC16 cells at a density of 10,000 per well were seeded in 96-well plates, incubated at 37 °C for 24 h, preincubated RosA for 24 h, and then treated with 1 μM Dox for 6 h. The cells were washed with PBS, and then we added 100 μL of a 1× binding buffer that contains 5 μL Annexin V-FITC (Molecular Probes, Eugene, OR, USA) and 5 μL Hochest 33342. Images were captured on a Leica microscope with a 20× objective and images were analyzed with ImageJ (http://rsb.info.nih.gov/ij/).

### 4.6. Ros Generation Determined by FACS

AC16 cells at a density of 400,000 per well were seeded in six-well plates and treated with RosA for 24 h, then treated with 1 μM Dox for 24 h. Cells were resuspended in 100 μL PBS, stained with 20 μL DCFH-DA, and incubated for 30 min at RT. Subsequently, cells were resuspended in 500 μL PBS and analyzed by BD caliber FACS (BD Bioscience, San Jose, CA, USA). ROS generation was measured by the fluorescence intensity.

### 4.7. ELISA Analysis

AC16 cells at a density of 40,000 per well were seeded in 24-well plates and treated with RosA for 24 h, then treated with 1 μM Dox for 24 h. Cell supernatant was collected and centrifugated at 3000× *g* for 5 min. Subsequently, the concentrations of NT-proBNP and IL6 were determined by Human NT Pro-BNP DuoSet ELISA kit (DY3604-05, R&D, Minneapolis, MN, USA) and Human IL-6 ELISA kit (3460-1HP-2, Nacka Strand, Sweden). The NT-proBNP and IL6 concentrations were normalized by total protein in cell supernatant, as determined by a Micro BCA Protein Assay Kit (Thermo Scientific, Rockford, IL, USA).

### 4.8. RNA Extraction and Quantitative PCR

Total RNA was isolated using an RNeasy Mini kit (Qiagen, Center Mainz, Germany) and quantified using a Nanodrop 2000 UV-visible spectrophotometer (Thermo Scientific, Wilmington, DE, USA). cDNA was prepared using 100‒500 ng/μL total RNA by reverse-transcription PCR using the PrimeScript RT reagent Kit (Takara, Mountain View, CA, USA), according to the manufacturer’s instructions. Real-Time PCR was performed on cDNA using SYBR Green probes. qPCR was performed on a 7500 Fast Real-Time PCR system (Applied Biosystems, Carlsbad, CA, USA) using SYBR^®^ Premix Ex Taq™ II (Takara, Mountain View, CA, USA). Fold changes in expression were calculated by the ΔΔCt method, using human GAPDH as an endogenous control for mRNA expression. All fold changes are normalized to the untreated control (Table 1).

### 4.9. Western Blotting

Western blot analysis was done according to standard protocols. The following antibodies were used: HDAC1 (1:1000, ab19845, Abcam, Cambridge, MA, USA), GATA4 (1:1000, ab84593, Abcam, Cambridge, MA, USA), cTnI (1:1000, ab47003, Abcam, Cambridge, MA, USA), GAPDH (1:4000, #5174, Cell Signaling, Danvers, MA, USA), Caspase 9 (1:1000, 66169-1-Ig, Proteintech, Rosemont, IL, USA), HO-1 (1:1000, 66743-1-Ig, Proteintech, Rosemont, IL, USA) and β-tubulin (1:4000, #86298, Cell Signaling, Danvers, MA, USA); secondary antibodies against mouse and rabbit were also used (1:8000, Abcam, Cambridge, MA, USA). Western blots were scanned and analyzed using the GelDoc system (Bio-Rad, Hercules, CA, USA).

### 4.10. Cheminformatics, Image Quantification, Pattern Recognition, and Statistical Analysis

Molecule structures were drawn using Open Babel (Department of Chemistry, University of Pittsburgh, Pittsburgh, PA, USA, releases 3.0.0). Image processing and analysis were performed using CellProfiler software (Broad Institute of Harvard and MIT, Cambridge, MA, USA, releases 3.1.9). The process project file is given in the Supplementary Materials. Data analysis, statistics, and pattern recognition algorithms are programmed using custom scripts in MATLAB (The Mathworks, Inc., Natick, MA, USA, version 2014b). Prism (GraphPad Software, San Diego, CA, USA, version 6.0) was used to perform the statistical analysis. All data were acquired via three independent replicate experiments. All data are presented as the mean ± SD (standard error), unless stated otherwise, and the statistical significance was evaluated using a Mann‒Whitney U test.

## Figures and Tables

**Table 1:** Primer sequences used for RT-PCR analysis.

Gene	Sequence (5′‒3′)	Product Length (bp)
HO-1 F	ACTGCGTTCCTGCTCAACAT	133
HO-1 R	GGGCAGAATCTTGCACTTTGT	
IL6 F	GGCACTGGCAGAAAACAACC	90
IL6 R	ACCAGGCAAGTCTCCTCATTG	
NPPB F	CTTTCCTGGGAGGTCGTTCC	86
NPPB R	GTTGCGCTGCTCCTGTAAC	
GAPDH F	CTTTGTCAAGCTCATTTCCTGG	133
GAPDH R	TCTTCCTCTTGTGCTCTTGC	

## Supplementary Materials

The following are available online at https://www.mdpi.com/1420-3049/25/4/836/s1, Figure S1: H9C2 cells treated with a series concentration of doxorubicin; Table S1: The performance of classifiers; Table S2: The results of natural compound screening.
